# Mikronährstoffe bei Säuglingen, Kindern und Jugendlichen – Bedeutung und Versorgungssituation

**DOI:** 10.1007/s00103-025-04129-y

**Published:** 2025-10-20

**Authors:** Anna-Kristin Brettschneider, Andrea Schlune, Clarissa Spiegler, Leonie Burgard, Regina Ensenauer

**Affiliations:** 1 Institut für Kinderernährung, Max Rubner-Institut (MRI), Bundesforschungsinstitut für Ernährung und Lebensmittel, Haid-und-Neu-Straße 9, 76131 Karlsruhe, Deutschland; 2 Institut für Ernährungsverhalten, Max Rubner-Institut (MRI), Bundesforschungsinstitut für Ernährung und Lebensmittel, Karlsruhe, Deutschland

**Keywords:** Nährstoffversorgung, Kritische Nährstoffe, Kindheit, Ernährungsmonitoring, Supplementierung, Nutrient supply, Critical nutrients, Childhood, Nutrition monitoring, Supplementation

## Abstract

Eine optimale Versorgung mit Mikronährstoffen ist für Säuglinge, Kinder und Jugendliche von großer Bedeutung und wird durch eine altersentsprechende, ausgewogene und abwechslungsreiche Ernährung gewährleistet. Daten zur Nährstoffversorgung dieser Altersgruppen liefern bundesweite Verzehrserhebungen wie die „Kinder-Ernährungsstudie zur Erfassung des Lebensmittelverzehrs“ (KiESEL-Studie) und die „Ernährungsstudie als KiGGS-Modul“ (EsKiMo II). Diese zeigen, dass ein erheblicher Anteil der Kinder und Jugendlichen in Deutschland nicht ausreichend mit wichtigen Mikronährstoffen wie Calcium, Eisen, Jod und Vitamin D versorgt ist. Zwar werden Nahrungsergänzungsmittel häufig verwendet, ihre Einnahme ist jedoch nur bei einem nachgewiesenen Mangel bzw. nur nach ärztlicher Beratung sinnvoll, um Überdosierungen zu vermeiden. Eine Supplementierung von einzelnen Nährstoffen (Fluorid, Vitamin D, Vitamin K) im Säuglingsalter wird regelhaft u. a. während der Kindervorsorgeuntersuchungen empfohlen. Auch bei bestimmten chronischen Erkrankungen oder besonderen Ernährungsweisen kann eine gezielte Supplementierung notwendig sein. Intensivierte Public-Health-Maßnahmen wie die Förderung einer gesunden Verpflegung in Kitas und Schulen sind nötig, um die Versorgung mit wichtigen Mikronährstoffen zu verbessern. Ein kontinuierliches Ernährungsmonitoring bei Kindern und Jugendlichen – wie es derzeit am Max Rubner-Institut aufgebaut wird – ist dabei essenziell, um Veränderungen in der Nährstoffversorgung zu erkennen und bei Defiziten gezielt entgegenzuwirken.

**Zusatzmaterial online:**

Zusätzliche Informationen sind in der Online-Version dieses Artikels (10.1007/s00103-025-04129-y) enthalten.

## Abstract (en)

An optimal supply of micronutrients is particularly important for infants, children and adolescents and is ensured by an age-appropriate, balanced and varied diet. Data on the nutrient intake of these age groups is provided by nationwide nutrition surveys such as the “Children’s Nutrition Survey to Record Food Consumption” (KiESEL) and the “Eating Study as a KiGGS Module” (EsKiMo II). These studies show that a considerable proportion of children and adolescents in Germany do not have an adequate supply of important micronutrients such as calcium, iron, iodine and vitamin D. Although dietary supplements are frequently used, they are only useful in the case of a proven deficiency and should only be taken after consulting a physician in order to avoid overdosing. Supplementation of individual nutrients (fluoride, vitamin D, vitamin K) is regularly recommended during well-child visits in infancy and early childhood. Targeted supplementation may also be necessary for certain chronic diseases or special diets. Intensified public health measures such as the promotion of healthy food in daycare centres and schools are necessary in order to improve the supply of important micronutrients. In addition, a continuous nutrition monitoring in children and adolescents—as is currently being established at the Max Rubner-Institut—is essential to detect changes in the nutrient intake and to counteract deficiencies in a targeted manner.

## Einleitung

Eine optimale Nährstoffversorgung ist in der Wachstumsphase von Säuglingen, Kindern und Jugendlichen hochrelevant und beinhaltet neben den energieliefernden Makronährstoffen auch eine bedarfsangepasste Zufuhr von Mikronährstoffen [1]. Zu den Mikronährstoffen zählen Vitamine (z. B. Vitamin B12, Vitamin D und Vitamin K) sowie Mineralstoffe (z. B. Calcium und Magnesium) und Spurenelemente (z. B. Eisen, Fluorid und Jod). Mikronährstoffe werden als essenzielle Nährstoffe vom Körper benötigt, um physiologische Funktionen aufrechtzuerhalten, einschließlich Wachstum, Entwicklung und Reproduktion, und sind u. a. für Stoffwechselprozesse, Immunsystem und zelluläre Funktionen unerlässlich [1, 2]. Ein deutlicher Mangel an Mikronährstoffen kann zu nährstoffspezifischen manifesten Erkrankungen, wie z. B. Rachitis bei Vitamin-D-Mangel, bzw. Symptomen, wie z. B. Knochenschmerzen, Muskelschwäche, Müdigkeit und erhöhte Infektanfälligkeit, führen [1]. Die Anforderungen an die Zufuhr von Vitaminen, Mineralstoffen und Spurenelementen sind bei Säuglingen, Kindern und Jugendlichen aufgrund der Bedeutung für zahlreiche biologische Entwicklungsprozesse besonders hoch. Die Mikronährstoffversorgung in der frühen Entwicklungsphase kann langfristig die Gesundheit im Erwachsenenalter beeinflussen [3].

In den ersten Monaten nach der Geburt ist ausschließliches Stillen insbesondere auch aufgrund der Zusammensetzung der Muttermilch die optimale Ernährung für Säuglinge. Diese dient dazu, den Nährstoffbedarf des Säuglings im ersten Lebenshalbjahr zu decken, und liefert die für eine gesunde Entwicklung wichtigen Mikronährstoffe [4, 5]. Ab dem Ende des ersten Lebenshalbjahres werden die Ernährungsbedürfnisse gesunder Säuglinge durch Milchnahrung allein nicht mehr gedeckt, da der Bedarf an Eisen, Zink und Vitamin B12 deutlich ansteigt [6]. Dieser Nährstoffbedarf wird über die Beikost zugeführt [7]. Spätestens mit Beginn des 7. Lebensmonats sollte ein erster Brei aus Gemüse, Kartoffeln und Fleisch oder Fisch eingeführt werden. Nach und nach folgen ein Milch-Getreide-Brei sowie ein Getreide-Obst-Brei [5]. Im weiteren Verlauf beginnt gegen Ende des ersten Lebensjahres der schrittweise Übergang zur Familienkost [7]. Auch nach Einführung von Beikost soll weiter gestillt werden, da Muttermilch aufgrund ihrer insgesamt hohen Nährstoffdichte noch bis ins 2. Lebensjahr hinein große Anteile des kindlichen Nährstoffbedarfs (u. a. Vitamin A, Vitamin B2, Calcium und Omega-3-Fettsäuren) abdecken kann [6].

Ab dem 2. Lebensjahr bis ins Erwachsenenalter bleiben die benötigten Nährstoffdichten, d. h. der Bedarf an Vitaminen und Mineralstoffen im Verhältnis zu den Empfehlungen für die Energiezufuhr, weitgehend konstant. Daher werden für die Lebensmittelauswahl für Kinder und Jugendliche verschiedener Altersgruppen dieselben Empfehlungen angewendet [8]. Bislang wird ab dem 1. Lebensjahr für Kinder und Jugendliche die Ernährung nach dem Prinzip der Optimierten Mischkost empfohlen, um die Referenzwerte für die Energie- und Nährstoffzufuhr für Kinder und Jugendliche zu erreichen [9, 10]. Die Optimierte Mischkost basiert auf einer abwechslungsreichen Ernährung mit Obst, Gemüse, Kartoffeln, Vollkornprodukten, maßvollem Verzehr von Milch und Milchprodukten, Fleisch, Fisch und Eiern. Öle und Fette sollten dabei sparsam eingesetzt und bevorzugt aus pflanzlichen Quellen gewählt werden [10]. Der Anteil der geduldeten Lebensmittel wie Süßigkeiten, Kuchen und salzige Snacks sollte unter 10 % der Gesamtenergiezufuhr liegen. In Deutschland erreichen diese Empfehlungen viele Kinder und Jugendliche jedoch nicht vollständig, sodass z. B. oft zu wenig Obst, Gemüse, Vollkornprodukte sowie Milch und Milchprodukte zu sich genommen werden, was die Versorgung mit wichtigen Mikronährstoffen beeinträchtigen kann [11–13]. Klinisch relevante Nährstoffmangelzustände bei ansonsten gesunden Kindern werden in Deutschland nur selten beschrieben. Dennoch besteht bei einzelnen Nährstoffen gemäß aktueller Verzehrstudien im Säuglings‑, Kindes- und Jugendalter im Mittel eine unzureichende Zufuhr [12, 14, 15]. Auf diese kritischen Nährstoffe sowie auf die Nährstoffe, deren Supplementierung zur Prophylaxe von Krankheiten regelhaft u. a. bei den Kindervorsorgeuntersuchungen empfohlen wird, wird im Folgenden fokussiert und hierbei auf die Daten der deutschlandweiten Ernährungserhebungen „Kinder-Ernährungsstudie zur Erfassung des Lebensmittelverzehrs“ (*KiESEL-Studie;* [16]) und „Ernährungsstudie als KiGGS-Modul“ (*EsKiMo II;* [12]) (Tab. 1) Bezug genommen.

**Tab. 1:** Repräsentative Ernährungsstudien bei Säuglingen, Kindern und Jugendlichen in Deutschland

Die „Kinder-Ernährungsstudie zur Erfassung des Lebensmittelverzehrs“ (*KiESEL;* [16]) und die „Ernährungsstudie als KiGGS-Modul“ (*EsKiMo II;* [12]) wurden als Modulstudien im Rahmen der 2. Welle der „Studie zur Gesundheit von Kindern und Jugendlichen in Deutschland“ (KiGGS Welle 2) bundesweit durchgeführt mit dem Ziel, detaillierte Daten zur Ernährung von Säuglingen, Kindern und Jugendlichen zu erheben, um die Nährstoffversorgung und Ernährungsgewohnheiten zu analysieren und bewerten	
**KiESEL**	**EsKiMo II**
Alter: 6 Monate bis 5 Jahre	Alter: 6 bis 17 Jahre
Stichprobenumfang: 1104 Teilnehmende	Stichprobenumfang: 2644 Teilnehmende
Erhebungszeitraum: 2014–2017	Erhebungszeitraum: 2015–2017

Zur Beurteilung der Nährstoffaufnahme der Säuglinge, Kinder und Jugendlichen im Alter von 6 Monaten bis 17 Jahren wurden die Referenzwerte der Deutschen Gesellschaft für Ernährung (DGE) und der Österreichischen Gesellschaft für Ernährung (ÖGE) (DGE/ÖGE-Referenzwerte; [9]) herangezogen. Für diese Bewertung wurde die Nährstoffaufnahme auf individueller Ebene relativ zu den alters- und geschlechtsspezifischen DGE/ÖGE-Referenzwerten berechnet. Für die EsKiMo II-Studie sind in dieser Arbeit die hierzu bereits publizierten Ergebnisse dargestellt [12]. Für die KiESEL-Studie wurden diese neu berechnet; ein Vergleich mit den Referenzwerten der Europäischen Behörde für Lebensmittelsicherheit (European Food Safety Authority – EFSA) ist in Burgard et al. publiziert [14, 15].

**Abb. 1:**
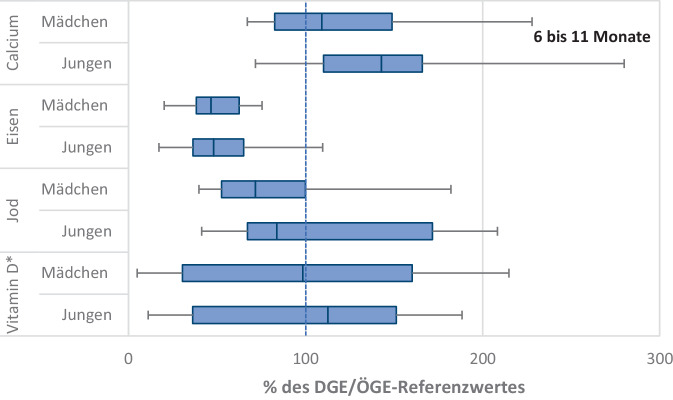
Verteilung kritischer Nährstoffe im Vergleich zu den DGE/ÖGE-Referenzwerten bei Säuglingen im Alter von 6 bis 11 Monaten aus der KiESEL-Studie. Ein Vergleich mit EFSA-Referenzwerten ist in Burgard et al. [15] publiziert. Dargestellt sind Box-Whisker-Plots mit Median sowie 25. und 75. Perzentile. Die „Whiskers“ geben den Bereich an, in dem die Werte zwischen dem 5. und 95. Perzentil liegen. DGE/ÖGE-Referenzwerte, Referenzwerte werden von der Deutschen Gesellschaft für Ernährung (DGE) und Österreichischen Gesellschaft für Ernährung (ÖGE) gemeinsam herausgegeben; EFSA, European Food Safety Authority; KiESEL-Studie, Kinder-Ernährungsstudie zur Erfassung des Lebensmittelverzehrs. Stern (*): inklusive Supplemente

Die Verteilung der jeweiligen Nährstoffe im Vergleich zu den DGE/ÖGE-Referenzwerten über die kindlichen Lebensphasen hinweg ist mit Box-Whisker-Plots dargestellt (Abb. 1, 2 und 3; Abb. Z1). Eine Übersicht der alters- und geschlechtsspezifischen DGE/ÖGE-Referenzwerte ist in Tab. 2 aufgeführt.

**Abb. 2:**
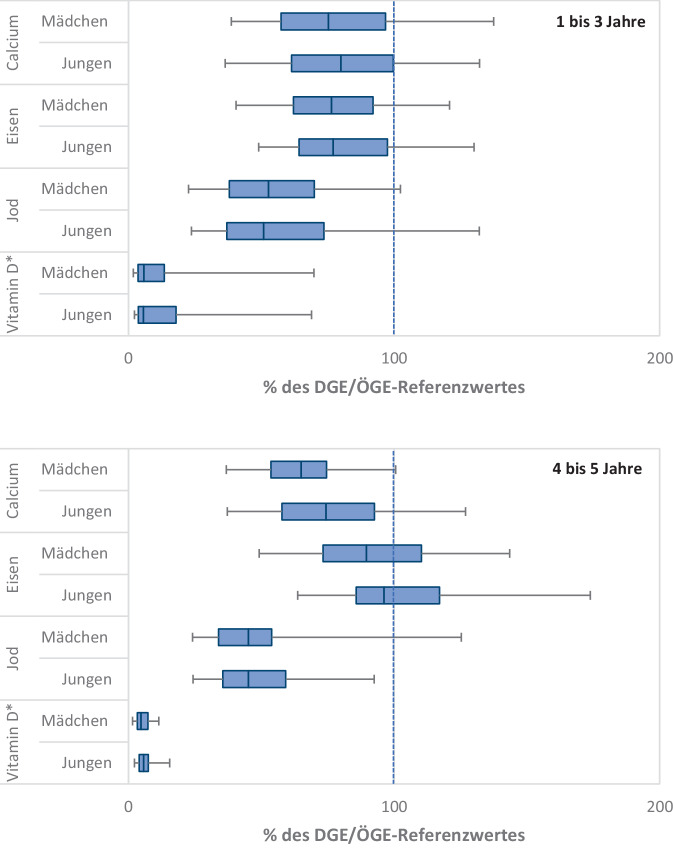
Verteilung kritischer Nährstoffe im Vergleich zu den DGE/ÖGE-Referenzwerten bei 1- bis 5-jährigen Kindern aus der KiESEL-Studie. Ein Vergleich mit EFSA-Referenzwerten ist in Burgard et al. [14] publiziert. Dargestellt sind Box-Whisker-Plots mit Median sowie 25. und 75. Perzentile. Die „Whiskers“ geben den Bereich an, in dem die Werte zwischen dem 5. und 95. Perzentil liegen. DGE/ÖGE-Referenzwerte, Referenzwerte werden von der Deutschen Gesellschaft für Ernährung (DGE) und Österreichischen Gesellschaft für Ernährung (ÖGE) gemeinsam herausgegeben; EFSA, European Food Safety Authority; KiESEL-Studie, Kinder-Ernährungsstudie zur Erfassung des Lebensmittelverzehrs. Stern (*): inklusive Supplemente

**Abb. 3:**
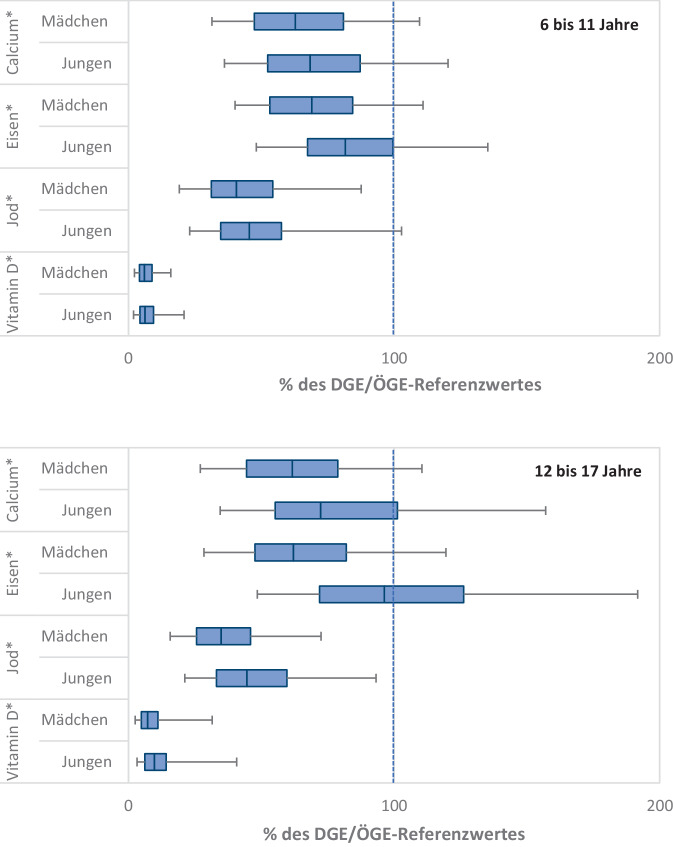
Verteilung kritischer Nährstoffe im Vergleich zu den DGE/ÖGE-Referenzwerten bei 6- bis 17-jährigen Kindern und Jugendlichen aus der EsKiMo II-Studie (eigene Abbildung nach Daten aus [12]). Dargestellt sind Box-Whisker-Plots mit Median sowie 25. und 75. Perzentile. Die „Whiskers“ geben den Bereich an, in dem die Werte zwischen dem 5. und 95. Perzentil liegen. DGE/ÖGE-Referenzwerte, Referenzwerte werden von der Deutschen Gesellschaft für Ernährung (DGE) und Österreichischen Gesellschaft für Ernährung (ÖGE) gemeinsam herausgegeben; EsKiMo II-Studie, Ernährungsstudie als KiGGS-Modul. Stern (*): inklusive Supplemente

**Tab. 2:** DGE/ÖGE-Referenzwerte für die Zufuhr ausgewählter relevanter Mikronährstoffe für Säuglinge, Kinder und Jugendliche [9]. Die Referenzwerte werden von der Deutschen Gesellschaft für Ernährung (DGE) und Österreichischen Gesellschaft für Ernährung (ÖGE) gemeinsam herausgegeben

Mikronährstoff	Referenzwert										
	0 bis < 4 Monate	4 bis < 12 Monate	1 bis < 4 Jahre	4 bis < 7 Jahre	7 bis < 10 Jahre	10 bis < 13 Jahre		13 bis < 15 Jahre		15 bis < 19 Jahre	
						m	w	m	w	m	w
Calcium [mg/Tag]	220	330	600	750	900	1100	1100	1200	1200	1200	1200
Eisen [mg/Tag]	0,3^a,b^	11	7	7	10	14	14^c^	11	16^d^	11	16^d^
Jod [µg/Tag]	40	80	100	120	140	180	180	200	200	200	200
Fluorid [mg/Tag]^1^	0,25	0,4	0,7	1,0	1,5	2,1	2,1	2,8	2,7	3,5	3,0
Vitamin D [µg/Tag]^2,3^	10	10	20	20	20	20	20	20	20	20	20
Vitamin K [µg/Tag]	4	10	15	20	30	40	40	50	50	70	60

^1^ Fluoridgesamtzufuhr aus Lebensmitteln, einschließlich fluoridierten Speisesalzes, und Getränken, inkl. Trink- und Mineralwasser, sowie Supplementen. Fluoridierte Zahnpflegeprodukte zur Kariesprävention tragen bei Verschlucken ebenfalls zur Gesamtzufuhr bei. Bei einer längeren Überschreitung der tolerierbaren Gesamtzufuhrmengen (etwa 0,1 mg/kg*Tag), besonders bei Kindern im Alter von 2–8 Jahren, ist mit einem erhöhten Risiko für das Auftreten von Zahnschmelzflecken (Zahnfluorose) zu rechnen

^2^ Bei fehlender endogener Synthese

^3^ 1 µg = 40 Internationale Einheiten (IE)

^a^ Ausgenommen Frühgeborene

^b^ Schätzwert

^c^ Bei frühzeitiger Menarche müssen menstruelle Eisenverluste ersetzt werden, wenn Menstruationsstärke und -zyklus bereits regelmäßig sind. Der physiologische Eisenbedarf entspricht in diesem Fall dem von prämenopausalen Frauen, wodurch sich eine empfohlene Zufuhr von 16 mg Eisen/Tag ergibt

^d^ Bei weiblichen Jugendlichen, die nicht menstruieren (u. a. aufgrund der Verwendung von oralen Kontrazeptiva, die durchgängig ohne Pause eingenommen werden), wird basierend auf den Werten für Männer eine empfohlene Zufuhr von 11 mg Eisen/Tag angegeben

## Kritische Nährstoffe in der Wachstums- und Entwicklungsphase

### Calcium

Calcium ist ein lebenswichtiger Mikronährstoff, der für viele wichtige Funktionen im Körper notwendig ist. Es unterstützt die Muskelkontraktion, die Weiterleitung von Reizen im Nervensystem und die Blutgerinnung. Besonders wichtig ist Calcium auch für den Aufbau von Knochenmasse und Zähnen. Bei Erwachsenen macht Calcium etwa 1–2 % des Körpergewichts aus, wobei über 99 % des gesamten Calciums im Körper in den Zähnen und Knochen gespeichert sind [17]. Daher ist der Calciumbedarf im Wachstumsalter erhöht. In der Pubertät ist das Knochenwachstum besonders intensiv. Der Calciumbedarf ist in dieser Phase am höchsten, weil die Knochenmasse deutlich zunimmt. Studien belegen, dass eine Calciumsupplementierung im Kindes- und Jugendalter mit einer Verbesserung des Knochenmineralgehalts und der Knochenmineraldichte einhergeht [18]. Ergebnisse der KiESEL-Studie zeigen, dass die Calciumaufnahme von Säuglingen im Alter von 6 bis 11 Monaten im Median über den DGE/ÖGE-Referenzwerten liegt (Abb. 1), während die Calciumzufuhr bei den 1‑ bis 3‑Jährigen und den 4‑ bis 5‑Jährigen im Median jeweils unterhalb der DGE/ÖGE-Referenzwerte liegt (Abb. 2). Auch die EsKiMo II-Studie zeigt, dass die Calciumaufnahmen von 6‑ bis 11-Jährigen sowie von 12- bis 17-Jährigen im Median jeweils unterhalb der DGE/ÖGE-Referenzwerte für die Nährstoffzufuhr liegen, unabhängig vom Geschlecht [12] (Abb. 3).

### Eisen

Eisen ist in der frühen Lebensphase nicht nur für die Blutbildung, sondern vor allem auch für die Entwicklung des Gehirns und des Nervensystems von essenzieller Bedeutung. Solide mechanistische Belege für die Rolle von Eisen bei der Gehirnentwicklung sowie Erkenntnisse aus Tierstudien deuten darauf hin, dass eine ausreichende Eisenversorgung während der Schwangerschaft und im Säuglingsalter von besonderer Relevanz für die neurologische Entwicklung ist [19]. Während der Schwangerschaft baut sich beim Neugeborenen in der Leber über die Plazenta ein Eisenspeicher auf. Ein verzögertes Abklemmen der Nabelschnur nach der Geburt führt zu einem höheren Hämoglobingehalt und stärkt die Eisenspeicher [6]. Da der physiologische Eisengehalt in der Muttermilch vergleichsweise gering ist, sind die kindlichen Eisenspeicher am Ende des 1. Lebenshalbjahres in der Regel aufgebraucht und der Eisenbedarf muss bei ausschließlich gestillten Kindern ab diesem Zeitpunkt über die Beikost abgedeckt werden. Als erste Beikostmahlzeit wird daher aktuell in Deutschland ein fleischhaltiger Brei empfohlen [5, 20]. Das darin enthaltene Häm-Eisen weist eine hohe Bioverfügbarkeit auf. Die Bioverfügbarkeit von Nicht-Häm-Eisen, wie es beispielweise in Getreide enthalten ist, kann durch die gleichzeitige Aufnahme von Vitamin C verbessert werden. Daher wird empfohlen, Getreidebreie im Rahmen der Beikost jeweils mit etwas Obst oder Obstsaft zuzubereiten. Die Ergebnisse der KiESEL-Studie zeigen jedoch Optimierungsbedarf, da die Eisenzufuhr bei Säuglingen im Alter von 6 bis 11 Monaten im Median unterhalb der DGE/ÖGE-Referenzwerte liegt (Abb. 1). Die Evidenz für den Einfluss eines Eisenmangels oder einer Eisensupplementierung auf die Entwicklung in der frühen Kindheit ist uneinheitlich [19]. Unter anderem gibt es Hinweise auf potenzielle negative Effekte einer ungezielten Eisensupplementierung, wie beispielsweise eine langfristige Beeinträchtigung der neurologischen Entwicklung bei Kindern ohne nachgewiesenen Eisenmangel [21]. Eine generelle Empfehlung zur Supplementierung besteht daher für Deutschland nicht.

Bei Kindern und Jugendlichen steigt der Eisenbedarf während der Wachstumsphase, um die erhöhte Zellteilung und Gewebeentwicklung zu unterstützen. Die KiESEL-Studie zeigt, dass die Eisenzufuhr bei Mädchen und Jungen im Alter von 1 bis 3 Jahren im Median unterhalb der DGE/ÖGE-Referenzwerte liegt, während sie bei 4‑ bis 5‑Jährigen im Median fast erreicht wird (Abb. 2). Ein unzureichender Eisenstatus kann bei Kindern und Jugendlichen zu Müdigkeit, Konzentrationsstörungen, verminderter Leistungsfähigkeit und Anämie führen. Geschlechterspezifische Unterschiede im Eisenbedarf treten insbesondere während der Pubertät auf. Mädchen benötigen aufgrund ihrer Menstruationsblutungen in der Regel mehr Eisen, um den durch die Blutung bedingten Eisenverlust auszugleichen und einem Eisenmangel vorzubeugen [1]. Die EsKiMo II-Studie zeigt, dass die mediane Eisenzufuhr insbesondere bei Mädchen im Alter von 6 bis 17 Jahren unterhalb der DGE/ÖGE-Referenzwerte liegt, während Jungen im Alter von 12 bis 17 Jahren die Zufuhrempfehlung fast erreichen [12] (Abb. 3).

### Fluorid

Für Fluorid sind keine „Fluoridmangelkrankheiten“ beschrieben. Jedoch ist Fluorid wichtig für den Knochenaufbau und die Zahngesundheit und stellt einen Schlüsselfaktor dar, um langfristig vor Karies zu schützen [22, 23]. Anstelle eines konkreten Bedarfs für die Zufuhr wird daher vielmehr ein Richtwert für eine angemessene Fluoridzufuhr angegeben, der aus der Beobachtung der optimalen Fluoridkonzentration im Trinkwasser beruht, bei der die geringste Karieshäufigkeit bei gleichzeitig minimalem Risiko für Dentalfluorosen besteht [24]. Fluorid wirkt sowohl bei topischer (z. B. über fluoridierte Zahnpflegeprodukte) als auch bei systemischer Anwendung (Erhöhung der Fluoridkonzentration im Plasma über z. B. Fluoridtabletten), indem es den Zahnschmelz widerstandsfähiger gegen Säureangriffe macht, die durch Bakterien im Mund entstehen. Daher ist eine ausreichende Fluoridzufuhr bereits beim Säugling wichtig. In Deutschland bestehen seit 2021 einheitliche Empfehlungen zur Fluoridprophylaxe zwecks Kariesprävention im Säuglings- und frühen Kindesalter [25] (Tab. 3). Eine Fluoridüberdosierung kann zu Skelett- oder Dentalfluorose in den bleibenden Zähnen führen [24]. Lebensmittel enthalten im Allgemeinen nur wenig Fluorid. Auch das Trinkwasser ist in Deutschland überwiegend fluoridarm (< 0,3 mg/l) und wird im Gegensatz zu anderen Ländern nicht mit Fluorid angereichert. Um die tolerierbare Tageshöchstmenge von Fluorid nicht zu überschreiten, wird empfohlen, dass Säuglinge, die ausschließlich oder überwiegend mit durch Wasser angerührte Muttermilchersatznahrung ernährt werden, kein Fluorid aus weiteren Quellen erhalten, wenn der Fluoridgehalt des verwendeten Wassers (Trinkwasser, Mineralwasser) ≥ 0,3 mg/l beträgt [25]. Auch bei Kindern ab dem Alter von 6 Jahren und Jugendlichen kann durch die Verwendung von Zahnpasta mit Fluoridgehalten zwischen 1000 ppm und 1450 ppm eine Reduktion der Karieshäufigkeit erreicht werden [26]. Daneben stehen für diese Altersgruppe auch weitere Fluoridierungsmaßnahmen wie Gelees oder Mundspüllösungen zur Verfügung, die insbesondere bei erhöhtem Kariesrisiko indiziert sein können.

**Tab. 3:** Empfehlungen zur Supplementierung von Mikronährstoffen im frühen Kindesalter

*Fluorid*	Zur Vorbeugung von Karies sollte von Geburt an täglich ein Kombinationspräparat mit 0,25 mg Fluorid und Vitamin D (siehe unten) eingenommen werden [25]. Diese Maßnahme kann über den Durchbruch des ersten Zahns hinaus bis zum Ende des 12. Lebensmonats fortgesetzt werden, vorausgesetzt, es wird keine Zahnpasta zum Zähneputzen verwendet oder fluoridfreie Zahnpasta (geringe Menge). Alternativ ist es möglich, bis zu 2‑mal täglich mit einer reiskorngroßen Menge einer Zahnpasta mit 1000 ppm Fluorid die Zähne zu reinigen und Vitamin D als Monopräparat zu geben. Ab dem zweiten Lebensjahr sollte die Fluoridprophylaxe durch die Verwendung einer fluoridhaltigen Zahnpasta, wie beschrieben, erfolgen.
*Jod*	In Deutschland wird aufgrund einer möglicherweise unzureichenden Jodzufuhr, insbesondere bei Säuglingen, die mit Muttermilch und selbstzubereiteter Beikost ernährt werden [29], die Empfehlung ausgesprochen, dass Stillende (nach vorheriger Jodanamnese) zusätzlich zur Verwendung von Jodsalz täglich etwa 100 μg Jod supplementieren [5]. Darüber hinaus wird für Säuglinge, die ausschließlich selbstgemachte Breie erhalten, eine tägliche Jodsupplementierung von 50 μg empfohlen [5].
*Vitamin D*	Die European Society for Paediatric Gastroenterology, Hepatology and Nutrition (ESPGHAN) empfiehlt allen Kindern von Geburt an bis zum Ende des 12. Lebensmonats täglich eine orale Gabe von 400 IE Vitamin D3 [35]. Gemäß der Ernährungskommission der Deutschen Gesellschaft für Kinder- und Jugendmedizin wird eine tägliche Supplementierung von 400–500 IE Vitamin D3 bis zum zweiten erlebten Frühsommer des Kindes empfohlen [36].
*Vitamin K*	Gesunden Neugeborenen wird eine orale Gabe von 3‑mal 2 mg Vitamin K in Form von Tropfen empfohlen [5, 37]. Die Vitamin-K-Gaben erfolgen jeweils am 1. Lebenstag, zwischen dem 3. und 10. Lebenstag (U2) und erneut zwischen der 4. und 6. Lebenswoche (U3) [37].

### Jod

Jod ist ein essenzielles Spurenelement mit einer Schlüsselrolle für die Schilddrüsenfunktion und hoher Relevanz für eine gesunde körperliche und geistige Entwicklung in den ersten Lebensmonaten. Gleichzeitig kommen Neugeborene mit physiologisch sehr geringen Jodspeichern zur Welt, obwohl der Jodbedarf im Säuglingsalter wegen des erhöhten Umsatzes von Schilddrüsenhormonen besonders hoch ist [27]. In Deutschland wird für Säuglinge in den ersten 4 Lebensmonaten eine tägliche Jodzufuhr von 40 µg empfohlen (Tab. 2), während in der Schweiz die WHO-Zufuhrempfehlung von 50 µg täglich gilt [9] und in den USA und Kanada 110 µg pro Tag empfohlen werden [27]. Die Unterschiede in den Empfehlungen spiegeln die begrenzte Evidenzgrundlage wider. Derzeit liegt auch keine eindeutige Evidenz für einen Zusammenhang zwischen leichtem bis moderatem Jodmangel und nachteiligen Gesundheitsoutcomes bei schwangeren und stillenden Frauen sowie bei Säuglingen und Kindern vor [28]. Hingegen gibt es Berichte über negative Auswirkungen eines maternalen Jodexzesses auf die neurologische Entwicklung von Säuglingen.

Es wurde gezeigt, dass es bei ausschließlicher Verwendung von Muttermilchersatznahrung und handelsüblichen kommerziellen Beikostprodukten rechnerisch zu einer Überschreitung der Jodzufuhr um bis zu 100 % kommen kann, während teilgestillte Säuglinge, die ausschließlich selbst hergestellte Beikost erhalten, weniger als 50 % der empfohlenen Jodzufuhr erreichen [29]. Die Jodversorgung von (ausschließlich) gestillten Kindern ist im Wesentlichen abhängig vom Jodgehalt in der Muttermilch und somit von der mütterlichen Jodzufuhr in den Stunden vor dem Stillen [27]. Stillenden Frauen wird daher zu einer Jodsupplementierung geraten, um den Jodgehalt ihrer Milch zu erhöhen [5] (Tab. 3). Darüber hinaus wird für Säuglinge, die ausschließlich selbst zubereitete Beikost erhalten, eine Supplementierung empfohlen [5]. Die Daten der KiESEL-Studie zeigen, dass die Jodzufuhr im Median bei Mädchen und Jungen im Alter von 6 bis 11 Monaten unterhalb des DGE/ÖGE-Referenzwertes liegt (Abb. 1).

In der weiteren Entwicklung von Kindern und Jugendlichen ist eine ausreichende Jodversorgung für eine normale körperliche Entwicklung, insbesondere für das Wachstum und die Gehirnentwicklung, entscheidend. Ein Jodmangel kann auch in der weiteren Kindheit und Jugend zu Störungen der kognitiven Fähigkeiten, verzögertem Wachstum und einer Schilddrüsenunterfunktion führen. Während der Wachstumsphasen steigt der Jodbedarf, um die erhöhte Hormonproduktion zu unterstützen. Jedoch wird über jodhaltige Lebensmittel in der Regel nicht ausreichend Jod aufgenommen, da durch die jodarmen Böden in Deutschland Getreide, Gemüse und Obst nur geringe Jodmengen enthalten [30]. Daher wird die Verwendung von jodiertem Speisesalz empfohlen [9]. Die Daten der KiESEL-Studie zeigen, dass die Jodaufnahme bei Mädchen und Jungen im Alter von 1 bis 5 Jahren im Median unterhalb der DGE/ÖGE-Referenzwerte liegt (Abb. 2). Auch bei der EsKiMo II-Studie liegt die mediane Jodzufuhr weit unterhalb der DGE/ÖGE-Referenzwerte (Abb. 3). Die tatsächliche Jodzufuhrmenge ist sowohl bei KiESEL als auch bei EsKiMo II vermutlich etwas höher, da der genaue Beitrag von jodiertem Speisesalz zur Jodzufuhr nicht erhoben wurde [12, 14]. Modellrechnungen zeigen jedoch, dass auch wenn die Verwendung von Jodsalz im Haushalt berücksichtigt wird, ein Risiko für eine unzureichende Jodzufuhr besteht [31]. Im Jahr 2023 startete das Bundesministerium für Landwirtschaft, Ernährung und Heimat daher die Informationsoffensive „Wenn Salz, dann Jodsalz“, um über die gesundheitliche Relevanz einer ausreichenden Jodzufuhr zu informieren und zu sensibilisieren [32].

### Vitamin D

Vitamin D hat eine besondere Bedeutung, da der Großteil des Bedarfs hauptsächlich durch die körpereigene Produktion in der Haut unter Einfluss von UVB-Strahlen bei Sonnenlicht gedeckt wird. Die Aufnahme über die Nahrung ist in der Regel nur von geringer Bedeutung. Vitamin D fördert über die Regulation des Calcium- und Phosphatstoffwechsels den Knochenaufbau und die Skelettmineralisierung. Eine ausreichende Versorgung mit Vitamin D ist insbesondere in der Phase von Wachstum und Knochenaufbau nötig und trägt dazu bei, eine optimale Knochenmineraldichte (*Peak Bone Mass)* zu erreichen, was wiederum hilft, späterer Osteoporose und Knochenbrüchen vorzubeugen [18]. Darüber hinaus übernimmt Vitamin D aber auch eine Vielzahl weiterer Funktionen, insbesondere in der Immunmodulation [33]. Da Muttermilch nur geringe Mengen an Vitamin D enthält und Säuglinge gemäß der Empfehlung [34] meist nicht der Sonne ausgesetzt sind (UV-Schutz), wodurch die körpereigene Vitamin-D-Produktion fehlt, wird in den ersten 1–2 Lebensjahren eine Supplementation empfohlen [35, 36] (Tab. 3). Als effektivste Form der Verbesserung des Vitamin-D-Status jenseits des 2. Lebensjahres wird von der Deutschen Gesellschaft für Kinder- und Jugendmedizin (DGKJ) ein regelmäßiger Aufenthalt im Freien mit Sonnenlichtexposition empfohlen (2-mal pro Woche zwischen 10 und 15 Uhr für 5–30 min mit unbedecktem Kopf sowie freien Armen und Beinen in den Monaten April bis September bei Kindern mit Hauttyp 2 und 3 unter der Prämisse der Sonnenbrandvermeidung; [36]). Eine prophylaktische Vitamin-D-Gabe in dieser Altersgruppe wird lediglich für Risikogruppen insbesondere in den Wintermonaten (500–1000 IE täglich) als sinnvoll angesehen. Diese umfassen Kinder und Jugendliche mit bestimmten chronischen Erkrankungen, sehr geringer Sonnenexposition und/oder dunkler Hautpigmentierung. Unter Berücksichtigung der Vitamin-D-Aufnahme aus Nahrungsergänzungsmitteln wurde bei Säuglingen der KiESEL-Studie im Median eine angemessene Zufuhr erreicht (Abb. 1). Bei den 1‑ bis 17-Jährigen zeigen dagegen sowohl die Daten der KiESEL-Studie als auch die der EsKiMo II-Studie, dass die Vitamin-D-Zufuhr über die Nahrung (inkl. Supplemente) im Median erheblich unterhalb der DGE/ÖGE-Referenzwerte liegt [12] (Abb. 2 und 3). Da der Großteil des Vitamin-D-Bedarfs durch die endogene Synthese in der Haut gedeckt wird, kann auf Basis dieser Daten allerdings nicht auf einen Mangel geschlossen werden.

### Vitamin K

Vitamin K spielt eine wichtige Rolle bei der Blutgerinnung, da es an der Bildung von Gerinnungsfaktoren beteiligt ist [37]. Aufgrund eines geringen Vitamin-K-Transports über die Plazenta kommen Neugeborene ohne ausreichenden Vitamin-K-Speicher zur Welt. Gleichzeitig ist die Darmflora, die zur Vitamin-K-Bildung beitragen kann, beim Neugeborenen noch nicht ausgebildet und in der Muttermilch ist nur wenig Vitamin K enthalten. In den ersten Lebenstagen besteht dadurch ein physiologischer Vitamin-K-Mangel, der mit dem Risiko von Blutungen einhergeht, die von Blutergüssen der Haut bis zu lebensbedrohlichen Gehirnblutungen reichen. Die Häufigkeit solcher Vitamin-K-Mangelblutungen wird historisch mit 0,25–0,5 % angegeben [38]. Besonders gefährdet sind gestillte Kinder und Kinder, deren Mütter Medikamente einnehmen, die den Vitamin-K-Stoffwechsel beeinflussen [39]. Zu diesen Medikamenten zählen Antikonvulsiva, Antibiotika, Antituberkulosemittel und Antikoagulanzien. Die Ernährungskommission der Amerikanischen Akademie für Kinderheilkunde empfahl erstmalig 1961 die postnatale Vitamin-K-Gabe zur Prophylaxe von Vitamin-K-Mangelblutungen [39]. In großen Surveillance-Studien, auch in Deutschland [40], wurde die Effektivität dieser Maßnahme bestätigt. Dass die prophylaktische Vitamin-K-Gabe eine weitgehende Verhinderung schwerwiegender Vitamin-K-Mangelblutungen ermöglicht, macht sie zu einer äußerst wichtigen Public-Health-Maßnahme weltweit. In Deutschland wird eine 3‑malige orale Gabe in den ersten Lebenswochen empfohlen (Tab. 3) [37]. Die KiESEL-Studie zeigt, dass Säuglinge im Alter von 6 bis 11 Monaten gut mit Vitamin K versorgt sind (Abb. Z1 im Onlinematerial). Auch bei Kindern und Jugendlichen zeigt sich, dass die mediane Vitamin-K-Zufuhr oberhalb der DGE/ÖGE-Referenzwerte liegt [12, 14].

## Besondere Ernährungssituationen in kindlichen Lebensphasen

Bestimmte chronische Krankheiten (z. B. Erkrankungen, die mit Malabsorption oder Maldigestion einhergehen, wie Zöliakie, Morbus Crohn oder zystische Fibrose bzw. Leber- oder Nierenerkrankungen) sowie Einschränkungen bei der Lebensmittelauswahl, z. B. im Rahmen von Allergien oder Lebensstil (vegetarische/vegane Ernährungsweise), gehen mit einem erhöhten Risiko für eine Mangelversorgung mit einzelnen (Mikro‑)Nährstoffen einher. Gerade in der vulnerablen Wachstums- und Entwicklungsphase bergen diese das Risiko ernster klinischer Folgen wie Gedeihstörungen oder irreversible neurologische Schädigungen. Dabei ist das Risiko für das Auftreten eines Nährstoffmangels umso höher, je stärker die Lebensmittelauswahl eingeschränkt wird. Allgemein kann eine ausgewogene vegetarische Ernährung mit dem Verzehr von Milch(‑produkten) und Eiern den Nährstoffbedarf auch im Säuglings‑, Kindes- und Jugendalter decken und Wachstum sowie eine altersentsprechende Entwicklung ermöglichen, wenngleich ein besonderes Augenmerk auf eine ausreichende Versorgung mit Eisen zu legen ist [41]. Eine vegane Ernährung, die komplett auf tierische Lebensmittel verzichtet, führt langfristig unabdingbar zu einem Mangel an Vitamin B12, eine Supplementierung ist daher zwingend notwendig. Auch auf die ausreichende Zufuhr der Mikronährstoffe Eisen, Zink, Jod und Calcium sowie Docosahexaensäure muss sehr genau geachtet werden, um gesundheitliche Konsequenzen durch eine unzureichende Nährstoffversorgung im Rahmen einer veganen Ernährungsweise zu vermeiden. Mit Blick auf das erhöhte Risiko für eine Nährstoffunterversorgung bzw. einen Nährstoffmangel und deren teilweise irreversiblen Konsequenzen hat die DGE bis 2020 eine vegane Ernährung für das gesamte Kindes- und Jugendalter nicht empfohlen [42]. Seit 2024 spricht sich die DGE aufgrund der eingeschränkten Datenlage weder eindeutig für noch gegen eine vegane Ernährung aus [43]. Auch die Ernährungskommission der DGKJ betont das beträchtliche Risiko einer Mangelversorgung mit Nährstoffen bei restriktiven Ernährungsformen [41]. Eine weitere Besonderheit stellt der Vitamin-B12-Mangel bei Neugeborenen dar, der durch eine unzureichende Zufuhr bei der Mutter in der Schwangerschaft entstehen kann. Für Deutschland wird geschätzt, dass etwa eines von 10.000 Neugeborenen davon betroffen ist [44]. Daher wird eine Früherkennungsuntersuchung in den ersten Lebenstagen im Rahmen des Neugeborenenscreenings als sinnvoll erachtet, um einen solchen Mangel frühzeitig zu erkennen und zu behandeln [45].

## Nahrungsergänzungsmittel

Die Einnahme von Nahrungsergänzungsmitteln in den kindlichen Lebensphasen wurde auch in der KiESEL- und EsKiMo II-Studie untersucht. Die KiESEL-Studie zeigte, dass 42 % der Kinder im Alter von 6 Monaten bis 5 Jahren in den letzten 12 Monaten vor der Befragung Nahrungsergänzungsmittel eingenommen hatten [46]. 35 % der Eltern gaben an, zur Prophylaxe Vitamin D zu supplementieren. Mit zunehmendem Alter sank generell die Supplementation mit Nahrungsergänzungsmitteln und somit auch die Vitamin-D-Supplementation: Im Alter von 6 bis 11 Monaten supplementierten 95 % der Kinder Vitamin D, während es bei 1‑jährigen Kindern 81 % waren. Ab einem Alter von 2 Jahren fiel der Anteil der Vitamin-D-supplementierten Kinder auf 42 % und bis zum Alter von 5 bis 6 Jahren sank er kontinuierlich auf 7 %. Der meistgenannte Grund für die Supplementation von Nahrungsergänzungsmitteln war die Empfehlung durch Kinderärztinnen und -ärzte.

Etwa 16 % der Jugendlichen im Alter von 12 bis 17 Jahren hatten laut EsKiMo II-Studie in den letzten 4 Wochen regelmäßig Nahrungsergänzungsmittel eingenommen, davon nahmen etwa 37 % Vitamine, 41 % Mineralstoffe und 46 % eine Kombination aus Vitaminen und Mineralstoffen ein [47]. Die am häufigsten verwendeten Vitaminpräparate enthielten Vitamin C, gefolgt von Vitamin D und Vitamin B12; bei den am häufigsten angegebenen Mineralstoffpräparaten waren es Magnesium, Zink und Eisen. Die hauptsächliche Motivation für die Einnahme von Vitamin- und Mineralstoffpräparaten war mit 59 % die Verbesserung der Gesundheit. Etwa ein Fünftel der Jugendlichen gab an, Nahrungsergänzungsmittel auf Empfehlung einer Ärztin oder eines Arztes einzunehmen, gefolgt von etwa 18 % der Jugendlichen, die eine Steigerung der körperlichen und geistigen Leistungsfähigkeit als Grund angaben.

Eine ausgewogene Ernährung bietet generell die beste Grundlage, um den Bedarf an Vitaminen, Mineralstoffen und anderen essenziellen Nährstoffen zu decken. Eine Supplementierung ist für gesunde Kinder daher nicht nötig. Ausnahmen bestehen in der Säuglingszeit und den ersten Lebensjahren, wie in Tab. 3 dargestellt für Fluorid, Jod, Vitamin D und Vitamin K. Weitere Nahrungsergänzungsmittel sollen nur nach ärztlicher Konsultation bei einem Mangel (z. B. bei Vorliegen einer Serumkonzentration unterhalb des Referenzbereichs) entsprechend der verordneten Dosierung eingenommen werden. Eine Mehraufnahme über den Bedarf hinaus hat nach aktuellem Kenntnisstand keinen zusätzlichen gesundheitlichen Nutzen [48]. Im Gegenteil kann eine übermäßige Zufuhr von Vitaminen und Mineralstoffen auch der Gesundheit schaden. Im Handel sind Nahrungsergänzungsmittel, die sich speziell an Kinder und Jugendliche richten, in vielfältigen Formen erhältlich. Um Eltern und Kinder gezielt anzusprechen, werden sie häufig auch als Bärchen, Säfte oder Fruchtgummis angeboten [49]. Gerade bei diesen Darreichungsformen besteht bei Kindern die Gefahr eines übermäßigen Verzehrs und somit einer Überdosierung, wie Fallbeispiele einer Vitamin-A-Überdosierung durch Kaubonbons zeigen [50].

## Fazit

Eine ausgewogene und abwechslungsreiche Ernährung für Säuglinge, Kinder und Jugendliche sorgt dafür, dass die altersentsprechenden Nährstoffbedürfnisse gedeckt werden, und stellt die Grundlage für eine optimale Mikronährstoffversorgung dar. Die Versorgung mit Mikronährstoffen wie Calcium, Eisen, Jod und Vitamin D ist jedoch bei einem großen Anteil der Kinder und Jugendlichen in Deutschland suboptimal und kann alters- und geschlechtsabhängig variieren. Bei bestimmten chronischen Erkrankungen oder besonderen Ernährungsweisen kann eine gezielte Supplementierung mit z. B. Vitamin B12 notwendig sein. Die Einnahme von Nahrungsergänzungsmitteln soll nach ärztlicher Beratung erfolgen, um Überdosierungen zu vermeiden. Public-Health-Maßnahmen, wie z. B. die Förderung einer gesunden Verpflegung in Kita und Schule oder die derzeit laufende Informationsoffensive „Wenn Salz, dann Jodsalz“ des Bundesministeriums für Landwirtschaft, Ernährung und Heimat, können dazu beitragen, das Bewusstsein für eine altersentsprechend optimierte und ausgewogene Ernährung zu stärken und die Versorgung mit kritischen Nährstoffen bei Kindern aller Altersklassen nachhaltig zu verbessern. Essenziell ist zudem ein regelmäßiges Ernährungsmonitoring bei Kindern und Jugendlichen, wie es derzeit am Max Rubner-Institut aufgebaut wird, um Veränderungen in der Nährstoffversorgung zu erkennen und bei Defiziten gezielt gegenzusteuern.

## Supplementary Information

Abb. Z1: Verteilung von Vitamin K im Vergleich zu den DGE/ÖGE-Referenzwerten bei Säuglingen im Alter von 6 bis 11 Monaten aus der KiESEL-Studie
