# Supplementary material for: Importance and supply of micronutrients in infants, children and adolescents
Source: Bundesgesundheitsblatt Gesundheitsforschung Gesundheitsschutz. 2025 Oct 20;68(11):1244–53. [Article in German] doi: 10.1007/s00103-025-04129-y (PMC12583406; doi:10.1007/s00103-025-04129-y)
Supplement: Supplementary file 1 — Abb. Z1: Verteilung von Vitamin K im Vergleich zu den DGE/ÖGE-Referenzwerten bei Säuglingen im Alter von 6 bis 11 Monaten aus der KiESEL-Studie [file 103_2025_4129_MOESM1_ESM.docx]

**Zusätzliches Onlinematerial**

**Abb. Z1:** Verteilung von Vitamin K im Vergleich zu den DGE/ÖGE-Referenzwerten bei Säuglingen im Alter von 6 bis 11 Monaten aus der KiESEL-Studie

Ein Vergleich mit EFSA-Referenzwerten ist in Burgard et al. [15] publiziert. Dargestellt sind Box-Whisker-Plots mit Median sowie 25. und 75. Perzentile. Die „Whiskers“ geben den Bereich an, in dem die Werte zwischen dem 5. und 95. Perzentil liegen. DGE/ÖGE-Referenzwerte, Referenzwerte werden von der Deutschen Gesellschaft für Ernährung (DGE) und Österreichischen Gesellschaft für Ernährung (ÖGE) gemeinsam herausgegeben; EFSA, European Food Safety Authority; KiESEL-Studie, Kinder-Ernährungsstudie zur Erfassung des Lebensmittelverzehrs

**6 bis 11 Monate**

.
